# Effect of community led total sanitation and hygiene (CLTSH) implementation program on latrine utilization among adult villagers of North Ethiopia: a cross-sectional study

**DOI:** 10.1186/s13104-019-4519-2

**Published:** 2019-08-02

**Authors:** Brhane Gebremariam, Kenfe Tsehaye

**Affiliations:** 1 School of Public Health, College of Health Sciences, Aksum University, Aksum, Ethiopia; 2 Department of Environmental and Occupational Health, College of Health Sciences, Aksum University, Aksum, Ethiopia

**Keywords:** Latrine utilization, CLTSH, Implementation, Ethiopia

## Abstract

**Objective:**

Absence of latrine remains a common public health problem in most of the Sub-Saharan Africa countries. A cross-sectional study was conducted to assess the effect of community-led total sanitation and hygiene approach implementation and associated factors among villages of Laelai Maichew District, Tigray, and North Ethiopia.

**Results:**

This study revealed that the rate of latrine use in the rural community of Laelai-Maichew district was about 46.8%. The majority, 71.1% of households in CLTSH implemented Villages and 93.5% of households in CLTSH non-implemented Villages did not have hand washing facility around their latrine. Community-led to total sanitation and hygiene non-implemented villages were 49% times less likely to utilize their latrine compared to those community-led total sanitation implemented villages [AOR = 0.51 95% CI (0.35, 0.75)]. Households owned latrines for two and above years were 1.5 more likely to utilize their latrine [AOR = 1.50 95% CI (1.21, 2.59)] than those of owning latrines for less than 2 years. In this study, latrine use rate was low. As a result, the local, national governmental and non-governmental organization should design programs to create behavioral changes on the community’s attitude towards latrine utilization.

**Electronic supplementary material:**

The online version of this article (10.1186/s13104-019-4519-2) contains supplementary material, which is available to authorized users.

## Introduction

World Health Organization 2017 report indicated that, in 2015 2.3 billion people who still lacked a basic sanitation service either practice open defecation (892 million) or use unimproved facilities such as pit latrines without a slab or platform, hanging latrines or bucket latrines (856 million) [1].

Rural communities such as, adolescent girls, and suffer most from inadequate hygiene and sanitation facilities because of vulnerability to social and economic aspects [2, 3]. The low hygiene and sanitation practice are considered as the main risk to development, affecting the nation’s improvement in well-being, educational status, and sex impartiality, societal and financial change universally. Besides, accessibility of better cleanliness and water availability are vital to halting the series of food shortage since it advances community health, initiation to effort and ability to attend school [3].

According to the Ethiopian Demographic and Health Surveys (EDHS) report of 2011 and 2016, the proportion of households with latrine facilities nationally increased from 55% in 2011 to 61% in 2016 [4, 5]. However, the progress was significantly lower than the stipulated national target of 100% coverage [6].

Community leads total sanitation and hygiene is new approach pioneered by Dr. Kamal Kar through the Village Education Resource Centre, which concentrates on empowering local people to analyze the extent and risk of environmental pollution caused by open defecation and to construct toilets without any external subsidies. The methodology is now being adopted in most regions of Ethiopia and elsewhere in Asia and Africa [7].

CLTSH implementation focuses on eradicating open defecation at a community level by bringing sustainable behavioral change leading to spontaneous and long term abandonment of OD practices and stimulating demand for latrines without any external hardware support [8–10].

Implementation of this approach began in different regions of the country; however, the effect of community-led total sanitation and hygiene approach on latrine use on implemented and non-implemented villages were, still undetermined, especially in our setup. Hence, this study was aimed at assessing the effect of CLTSH implementation on latrine utilization in adult villager’s in Laelai Maichew District, Tigray, Ethiopia.

## Main text

### Methods and materials

#### Study area

Ethiopia is a home of various ethnicities. It has nine regions and two administrative towns. The state of Tigray shares common borders with Eritrea in the north, the State of Afar in the east, the State of Amhara in the south, and the Republic of Sudan in the west. There are 52 districts with 792 kebeles (the smallest administrative unit in Ethiopia) in the region. The total population of the region is estimated as 5,128,532 population [11]. Tigray regional state has two specialized hospitals, 16 general hospitals, 11 primary hospitals, 218 health centers, and 668 health posts. Apart from this, the region has four universities with important health science fields of study. The greatest part of the community (80%) are agriculturalists [12]. In this region rate of open defecation is 35.6% which is slightly higher than the national standard [13] and in 2011 the latrine coverage of the region were 87% though utilization was minimal (34%) [14].

#### Study design and period

The community-based cross-sectional study design was conducted in Laelai Maichew, District from February 2017 to April 2017 in Tigray, Northern Ethiopia.

#### Study population

The study population was randomly selected households with available private latrine.

#### Sample size determination and procedure

Single population proportion formula was used to determine the sample size in this study assuming latrine use rate 50%, 95% confidence level, Design effect of 2, 5% margin of error and 10% of non-response rate.

$${\text{n}} = {\text{DE}}\frac{{\left( {{\text{Z}}a/2} \right)^{2} {\text{P}}\left( {1 - {\text{P}}} \right)}}{{{\text{d}}^{2} }} = {\text{DE}}\frac{{\left( {1.96} \right)^{2} 0.5\left( {1 - 0.5} \right)}}{{\left( {0.05} \right)^{2} }} = 2\left( {384} \right) = 768$$

Since the expected total number of households in the district is less than 10,000 (which is 8253) we use finite population correction formula as shown below:-${\text{nf}} = 768/\left( {1 + \left( {{\text{n}}/{\text{N}}} \right)} \right) = {\text{nf}} = \left( {1 + \left( {768/8253} \right)} \right) = 705,$ then considering 10% non-response rate a total of 776 study subjects was included in the study.

The multistage sampling procedure was used, where the Villages (smallest rural residential area in Ethiopia context) first divided into CLTSH implemented and non-implemented, then three Villages were selected from each total Villages by lottery method. Then, to draw a sampling frame the total number of households in the Villages were obtained from the respected Health Extension workers of each village. The sample size was allotted to each selected village by probability proportional to size sampling method. Using systematic random sampling (every ninth household) the final sample size included in the study was 776 [15].

### Data collection method and instrument

An interviewer-administered structured questionnaire was prepared after reviewing articles from previously done studies to collect data. Whereas, for latrine utilization, an observational checklist was used to assess the utilization rate.

#### Data quality control

Training of data collectors, questionnaire pre-test and translation to local language were made to assure the quality of the data. Consistent and routine monitoring of the data collectors were also made to maintain the quality of the data collection process and completeness and consistency of the data followed by feedback.

### Variables

#### Outcome variable

Latrine utilization.

#### Explanatory variable

- Socio-economic characteristics: Age, Sex, Marital status, Educational status, Monthly income, Presence of school children.
- Behavioral characteristics: Frequency of latrine use, Observable feces in the compound and latrine, Disposal means of children feces.
- Environmental characteristics: Place of defecation, Time of latrines construction, superstructure, presence of hand washing facilities near the latrine, the distance of latrine from the house and dwelling.

#### Data processing and analysis

Data were coded, checked, cleaned and corrected for errors and entered into SPSS version 20.0 and analyzed. Bivariate logistic regression was used to identify the predictor variables associated with the outcome variable. The odds ratio was computed to show the strength of the association of the explanatory variables and the dependent variables. Statistical significance tests were assured using odds ratio at the cut-off value of 95% CI and p < 0.05.

#### Ethical consideration

Ethical clearance and permission letter were obtained from Aksum University College of Health Sciences Ethical committee and from the district health office, respectively. Verbal and written consent was also obtained from the participants explaining the purpose of the study.

### Results

#### Demographic characteristics of participants

A total of 776 participants, from CLTSH, implemented and CLTSH non-implemented villages completed the study giving a response rate of 100%. The average age of the respondents were 42 ± 5.5 (mean ± SD) and the average family size was 5.5.

Around 249 (64.2%) of the respondents in CLTSH implemented and 259(66.8%) of the respondents in CLTSH non-implemented villages were male respondents. Two hundred ten (54.1%) respondents from CLTSH implemented and 197(50.8%) respondents from non-implemented villages had an age group of above forty years, respectively. 187(47.7%) of the respondents from CLTSH implemented and 216(55.7%) of the respondents in non-implemented villages had 1–4th-grade educational level, respectively. Above 90% of the respondents in both CLTSH implemented and non-implemented villages were headed by farmers (Table 1).

**Table 1:** Socio-economic conditions of the households in the study area, February-April, 2017

Variables	CLTSH implemented	
	Yes (%)	No (%)
Sex		
Male	249 (64.2)	259 (66.8)
Female	139 (35.8)	129 (33.2)
Age		
26–40	178 (45.8)	191 (49.3)
> 40	210 (54.1)	197 (50.8)
Marital status		
Married	349 (89.9)	384 (99)
Single	13 (3.4)	3 (0.8)
Widowed	11 (2.8)	0 (0)
Divorced	15 (3.9)	1 (0.3)
Educational level		
Illiterate	187 (48.2)	154 (39.7)
Grade 1–4th	185 (47.7)	216 (55.7)
Grade 5–12th	13 (3.4)	18 (4.6)
Diploma and above	3 (0.8)	0
Family head		
Farmer	361 (93)	382 (98.5)
Daily laborer	12 (3.1)	2 (0.5)
Merchant	15 (3.9)	4 (1)
Presence of school children		
No	67 (17.3)	108 (28.1)
Yes	320 (82.7)	276 (71.9)
Family size		
1–3	44 (11.3)	10 (2.6)
4–6	246 (63.4)	250 (64.4)
> 7	98 (25.3)	128 (33)

#### Latrine utilization

In this study, about 145(54.9%) of the CLTSH implemented and 89 (38.7%) of CLTSH non-implemented villages declared that they utilized their latrine which gives the overall utilization rate of 46.8% (Table 2).

**Table 2:** Environmental and behavioral characteristics of households of the study area, February–April, 2017

Variables	CLTSH Implemented	
	Yes (%)	No (%)
Latrine availability (n = 776)		
No	124 (32)	158 (40.7)
Yes	264 (68)	230 (59.3)
Place of defecation		
Open field	69 (55.6)	77 (48.7)
Other	55 (44.4)	81 (51.3)
Service year of latrine (years)		
< 2	79 (29.9)	66 (28.7)
> 2	185(70.1)	164 (71.3)
Maintenance of latrine required		
Yes	129 (49)	129 (50)
No	134 (51)	101 (49)
Presence of squat hole cover		
No	162 (61.4)	131 (57)
Yes	102 (38.6)	99 (43)
Presence of hand washing facility near latrine		
Present	11 (4.2)	15 (6.5)
Absent	253 (95.8)	215 (93.5)
Distance of latrine to dwelling room (m)		
<6	40 (15.2)	18 (7.8)
6–10	188 (71.2)	195 (84.8)
> 10	36 (13.6)	17 (7.4)
Distance of home from kebelle		
Nearest to medium	249 (64.2)	223 (57.5)
Far	139 (35.8)	165 (42.5)
Frequency of visit by local leaders per week		
Once	38 (9.8)	83 (21.4)
Twice	148 (38.1)	146 (37.6)
Three and above	202 (52.1)	159 (41.0)
Latrine utilization (n = 494)		
Yes	145 (54.9)	89 (38.7)
No	119 (45.1)	141 (61.3)
Hand washing practice after using the toilet (= 776)		
Yes	241 (62.1)	211 (54.4)
No	147 (37.9)	177 (45.6)
Water consumption per person per liter (= 776) (l)		
≤ 10	132 (34)	101 (26)
> 10	256 (66)	287 (74)
Presence of feces around the latrine (n = 494)		
Yes	92 (35.1)	106 (45.7)
No	170 (64.9)	126 (54.3)
Presence of fresh excreta in the compound (n = 494)		
Yes	131 (33.8)	207 (53.4)
No	257 (66.2)	181 (46.6)

#### Environmental and behavioral conditions

Finding of this study showed that 264 (68%) households in community-led total sanitation and hygiene implemented Villages and 230 (59.3%) of households in community-led total sanitation and hygiene non-implemented Villages owned latrine. 68(55.3%) and 77(48.7%) of households in community-led total sanitation and hygiene implemented and non-implemented Villages with no available toilet at home, practices open defecation, respectively.

Additionally, 249 (64.2%) of households in community-led total sanitation and hygiene implemented Villages and 221 (57.9%) households in community-led total sanitation and hygiene non-implemented Villages had near to medium distance to their respected Kebelle.

One hundred forty-seven (37.9%) of household in community-led total sanitation and hygiene implemented Villages and one hundred seventy-seven (45.6%) of household in community-led total sanitation and hygiene non-implemented Villages do not wash their hand after using the toilet. Regarding hand washing practice of households, ninety-five (39.4%) in CLTSH implemented Villages and eighty-one (38.4%) in CLTSH non-implemented Villages always wash their hands. Furthermore, one hundred seventy (64.9%) of household in CLTSH implemented Villages and one hundred twenty-six (54.3%) of households in community-led total sanitation and hygiene non-implemented Villages had fresh excreta around their latrine (Table 2).

#### Factors associated with latrine utilization

Age, marital status, service year of the latrine, the status of CLTSH implementation and distance of latrine from the house were independent predictors of latrine utilization in the bivariate logistic regression model. Whereas, in multivariate logistic regression model age and marital status did not significantly associate with the outcome variable. CLTSH non-implemented villages were 0.51 times less likely to utilize their latrine compared to those community-led total sanitation implemented villages [AOR = 0.51 95% CI (0.35, 0.75)]. Households owned latrines for 2 and above years were 1.5 more likely to utilize their latrine [AOR = 1.50 95% CI (1.21, 2.59)] than those of owning latrines for less than 2 years. The odds of utilizing latrine in households with less than 10-m latrine distance from dwelling were 3.24 times higher than [AOR = 3.24 95% CI (1.46, 7.18)] those who have greater than a 10-m distance in the study (Table 3).

**Table 3:** Final regression model of the factors related to latrine utilization in the study area, February–April 2017

Variables	Category	Latrine use		COR (95% CI)	AOR (95% CI)
		Yes	No		
		Number (%)	Number (%)		
Age	18–25	4 (80)	1 (20)	0.22 (0.03, 2.02)*	0.21 (0.02, 2.15)
	26–40	97 (46.9)	110 (53.1)	1.01 (0.71, 1.45)	0.75 (0.45, 1.26)
	> 40	133 (47.2)	149 (52.8)	1	1
Marital status	Single	6 (66.7)	3 (33.3)	2.50 (0.19, 32.19)	3.45 (0.39, 30.44)
	Married	219 (47)	247 (53)	5.64 (0.65, 45.6)	2.57 (0.19, 35.1)
	Divorced or separated	4 (30.8)	9 (69.2)	11.25 (0.97, 130.22)	8.1 (0.68, 95.8)
	Widowed	5 (83.3)	1 (16.7)	1	1
CLTSH implemented	No	89 (38.7)	141 (61.3)	0.52 (0.36, 0.74)*	0.51 (0.35, 0.75)**
	Yes	145 (54.9)	119 (45.1)	1	1
Year of latrine construction (years)	≥ 2	172 (49.3)	177 (50.7)	1.30 (0.88, 1.92)*	1.50 (1.21, 2.59)*
	< 2	62 (42.8)	83 (57.2)	1	1
The distance of latrine from dwelling (m)	< 10	21 (36.2)	37 (63.8)	3.15 (1.45, 6.85)*	3.24 (1.46, 7.18)*
	6–10	179 (46.7)	204 (53.3)	2.04 (1.12, 3.70)*	1.70 (0.92, 3.17)
	Above 10	34 (64.2)	19 (35.8)	1	1

* Significantly associated at p < 0.05

** Significantly associated at p < 0.001

### Discussions

This study revealed that the overall latrine utilization was 46.8%, 95% CI (41.2–52.1%). This was to some extent lower than a study conducted in Hulet Ejju Enessie district, East Gojjam Zone, Amhara Region 60.7% [16], Gulemokada, 57.3% [17] and Alaba and Mirab Abaya districts 93%, Ethiopia [18]. The main cause might be due to lack of disorganized supervision mechanism and lack of focus by government politicians on environmental sanitation in the study site. Another reason for the low utilization of latrines can be explained that health extension workers promote the construction of latrine rather than utilization and less active in teaching proper latrine utilization.

The odds of utilizing latrine was 1.5 times higher on households who own the latrine for more 2 years compared to those who own less than 2 years [AOR = 1.5 95% CI (1.21-2.59)]. This may be attributed to the perception of the community to gain immediate health benefit like cleanliness and reduction of fly breeding. However, this has not any consequence on households which did not implement community-led total sanitation and hygiene.

In this study CLTSH non-implemented villages were 49% less likely to utilize their latrine than their counterparts [AOR = 0.51 95% CI (0.35, 0.75)]. This may be due to knowledge and awareness of the community in the study area about CLTSH which was incompletely and disorganized given by some local NGO’s like Relief Society of Tigray (REST). This study was in similar to a study done in Bahir Dar Zuria, Ethiopia [19].

The findings of this study also presented that of the households without latrine, above half (55.3%) of households in community-led total sanitation and hygiene implemented and 48.7% of households in non-implemented Villages were defecate openly in the study time. This was comparable to a study conducted in Jimma zone, Kersa District, Ethiopia [20].

In this study, almost half of the latrines in both CLTSH implemented (49%) and (50%) in CLTSH non-implemented Villages needs maintenance. The presence of a handwashing facility near latrine encourages the users to wash their hands after latrine use [20]. Ninety-six percent in CLTSH implemented and 93.5% in CLTSH non-implemented Villages have no hand washing facility near their latrine. A finding from Gulomekada District [17] noted that the presence of school children was associated with latrine utilization, whereas in our study this variable was not significantly associated.

### Conclusions

Latrine utilization rate in this study was found relatively low compared to the national standards. Therefore, even though the latrine use seems comparable both in the CLTSH implemented and non-implemented Villages, regional health bureau, federal ministry of health and non-governmental organizations should exert their unreserved and coordinated efforts on creating awareness on the community to utilize their latrine beyond concentrating on construction of latrines.

## Limitation

- Due to the inconsistent and disorganized implementation of the CLTSH approach in the community, there may be leakage of information between the implemented and non-implemented villages which may have over reported or incomplete report.

## Additional file

**Additional file 1.** Minimal data set included in the manuscript.

## Data Availability

All pertinent data was in the manuscript. The datasets investigated during this study was available from the Corresponding author on reasonable request (Additional file 1).

## Abbreviations

CLTSH: community led total sanitation and hygiene
EDHS: Ethiopian Demographic and Health Surveys
ODF: open defecation free
UNICEF: United Nation’s International Children’s Emergency Fund
